# An *Agrobacterium tumefaciens* Strain with Gamma-Aminobutyric Acid Transaminase Activity Shows an Enhanced Genetic Transformation Ability in Plants

**DOI:** 10.1038/srep42649

**Published:** 2017-02-21

**Authors:** Satoko Nonaka, Tatsuhiko Someya, Sha Zhou, Mariko Takayama, Kouji Nakamura, Hiroshi Ezura

**Affiliations:** 1 Gene Research Center, Faculty of Life and Environmental Sciences, University of Tsukuba, 1-1-1 Tennodai, Tsukuba-shi, Ibaraki 305-8572, Japan

## Abstract

*Agrobacterium tumefaciens* has the unique ability to mediate inter-kingdom DNA transfer, and for this reason, it has been utilized for plant genetic engineering. To increase the transformation frequency in plant genetic engineering, we focused on gamma-aminobutyric acid (GABA), which is a negative factor in the *Agrobacterium*-plant interaction. Recent studies have shown contradictory results regarding the effects of GABA on *vir* gene expression, leading to the speculation that GABA inhibits T-DNA transfer. In this study, we examined the effect of GABA on T-DNA transfer using a tomato line with a low GABA content. Compared with the control, the T-DNA transfer frequency was increased in the low-GABA tomato line, indicating that GABA inhibits T-DNA transfer. Therefore, we bred a new *A. tumefaciens* strain with GABA transaminase activity and the ability to degrade GABA. The *A. tumefaciens* strain exhibited increased T-DNA transfer in two tomato cultivars and *Erianthus arundinacues* and an increased frequency of stable transformation in tomato.

*Agrobacterium* is a genus of gram-negative bacteria that includes strains that are able to transfer genes that cause tumors (*Agrobacterium tumefaciens* or *A. vitis*) or hairy root (*A. rhizogenes*). *A. tumefaciens*, which induces crown gall disease in plants at the junction of the root and shoot, has been thoroughly studied, and the molecular mechanisms of gene transfer by this species have been elucidated^1^. *A. tumefaciens* harbors the Ti plasmid, which includes *vir* genes and transfer DNA (T-DNA) regions. The T-DNA regions contain oncogenic genes, such as indole-3-acetic acid (IAA), cytokinin and opine synthesis genes. Phenolic compounds and sugars exuded from the plant root induce *vir* gene expression^2^,^3^,^4^,^5^,^6^, after which the T-DNA region is excised by VirC and VirD. The excised single-stranded T-DNA forms a T-DNA complex with VirD and VirE. This T-DNA complex is introduced into plant cells via the type IV secretory system and then enters the plant nuclei through the intercellular transport system. The VirD and VirE proteins are subsequently stripped off, and the T-DNA is integrated into the plant nucleus. Expression of the T-DNA region integrated into the plant genome causes crown gall disease.

Although *A. tumefaciens* causes plant disease, its unique ability to transfer DNA presents the possibility that useful traits can be introduced into crops^7^,^8^, indicating potential for use in plant genetic engineering. To adapt the bacterium for plant genetic engineering, many efforts have been made to remove the oncogenic abilities of *A. tumefaciens* and invent a binary vector system^9^,^10^,^11^,^12^, representing the first step in adaption for plant genetic engineering. The next step is to expand the host range and increase the transformation frequency. Upregulation of *vir* gene expression is an effective strategy for broadening the host range of the bacterium and increasing transformation. Application of *vir* gene inducers^2^,^3^,^4^,^5^,^6^, utilization of super-binary vectors^13^,^14^,^15^ and employing a ternary transformation system^16^ improve the transformation efficiency. Depression of negative effectors of *Agrobacterium*-plant interactions is also effective. For instance, the phytohormone ethylene is a negative factor in *Agrobacterium*-plant interactions^17^,^18^,^19^,^20^. The ability of *A. tumefaciens* strains to reduce ethylene evolution from plants increases the transformation frequency^21^,^22^,^23^,^24^.

Gamma-aminobutyric acid (GABA) is a negative regulator of *Agrobacterium*-plant interaction through the quorum-sensing (QS) signal, which induces horizontal transfer of the Ti plasmid^25^,^26^. GABA imported into *A. tumefaciens* moderates crown gall disease symptoms via degradation of the QS signal. In tumors, accumulation of opines induces a QS signal, which enhances conjugation of the Ti plasmid. High accumulation of GABA depresses the QS signal, resulting in inhibition of Ti plasmid conjugation^27^, and then GABA plays a role in tumor at a later stage of the *Agrobacterium*-plant interaction. In a GABA-rich tobacco line, crown gall disease symptoms are less severe than in the wild type^25^. An *atu2422*-defeated *A. tumefaciens* strain, which lacks the ability to take up GABA, was shown to cause severe symptoms in tobacco^26^. However, no significant differences in *vir* gene expression or T-DNA transfer are observed between the *atu2422*-defeated strain and the wild-type strain^26^. From these results, it was concluded that GABA controls crown gall disease through a pathway independent of *vir* gene expression and T-DNA transfer. In contrast, a recent study showed that the expression of *vir* genes decreases in *her1* (an *Arabidopsis thaliana* mutant line), which shows higher accumulation of GABA^28^. The accumulated GABA suppresses *vir* gene expression, which is essential for T-DNA transfer. Therefore, the accumulation of high GABA levels may inhibit T-DNA transfer via *vir* gene suppression.

In this study, we examined whether GABA affects T-DNA transfer using a low-GABA tomato line that we produced and previously characterized^29^. Additionally, we bred a new *A. tumefaciens* strain showing GABA transaminase activity (GabT) and the ability to degrade GABA. Although the *atu3300* gene, which exhibits high similarity to the GABA transaminase gene, is located in the linear chromosome of *A. tumefaciens* strain C58^30^, activity of this gene has not yet been reported^31^. We then cloned the GABA transaminase gene (*gabT*) from *Escherichia coli* K12 32 and introduced it into *A. tumefaciens*. The ability of T-DNA transfer in the new *A. tumefaciens* strain was evaluated in two tomato cultivars (‘Micro-Tom’ and ‘Moneymaker’) and the grass *E. arundinaceus*, which is a potential biomass plant, and stable transformation was examined in tomato (‘Micro-Tom’).

## Results

### Inoculation of *A. tumefaciens* stimulates GABA accumulation during co-cultivation

**Figure 1:**
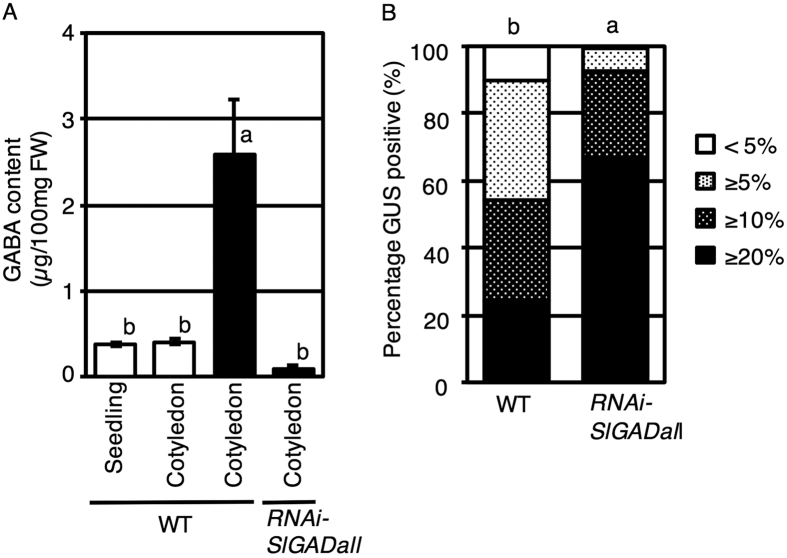
GABA inhibits *Agrobacterium*-mediated T-DNA transformation. (**A**) GABA content in intact seedlings and cotyledon segments of Micro-Tom. White and black indicate un-inoculated and inoculated, respectively. Bars indicate the standard deviation (n = 3). Different letters indicate significant differences by Tukey’s test (*P* < 0.01). (**B**) Classification of GUS-stained cotyledon explants. GUS-stained tomato cotyledons were categorized based on the stained area, as follows: 20% or more of the area, 10% or more, 5% or more, or less than 5%. The frequency of each category of GUS-stained tomato explants is shown. Bacterial strains exhibiting significant differences (Student’s *t*-test and Kruskal-Wallis test; *P* < 0.01, n = 80) are indicated with different letters. WT and RNAi refer to the non-transgenic line and the RNAi-SlGADall line, respectively. In GAD RNAi lines, the expression of *GAD1, GAD2,* and *GAD3* was much lower than in WT.

To evaluate the effect of *A. tumefaciens* inoculation on GABA accumulation, the cotyledon segments from 7-day-old tomato seedlings were used, and both un-inoculated and inoculated segments were prepared. After 3 days of co-cultivation, GABA accumulation in tomato (Micro-Tom) cotyledon segments was measured. In tomato cotyledon segments inoculated with *A. tumefaciens* (black bar, WT, Cotyledon), the GABA level was 5 times higher than in un-inoculated segments (white bar, WT, Cotyledon) (Fig. 1A). To determine whether wounding caused GABA accumulation, intact seedlings (white bar, WT, Seedling) and tomato cotyledon segments (white bar, WT, Cotyledon) were compared. In the un-inoculated treatment, the GABA content in the cotyledon segments was the same as in the intact seedlings. These results indicate that GABA accumulation was stimulated by *A. tumefaciens* infection, rather than that by wound stress.

### GABA affects T-DNA transformation in tomato

If accumulation of GABA during co-cultivation inhibits T-DNA transfer, the frequency of T-DNA transfer should be increased in low-GABA plants. We investigated whether GABA affects T-DNA transfer using the GAD RNAi transgenic *S. lycopersicum* cv. Micro-Tom (*RNAi-SlGADall*)^29^. In the *RNAi-SlGADall* line, the expression levels of GAD genes (*GAD1, GAD2*, and *GAD3*) involved in GABA synthesis were reduced, and the accumulation of GABA was low compared with the non-transgenic line (Fig. 1A). To evaluate the T-DNA transfer efficiency, 80 tomato cotyledon segments were prepared from 7-day-old seedlings and inoculated with *A. tumefaciens* GV2260 (pIG121-Hm). The *uidA* gene was used as an indicator of T-DNA transfer. After 3 days of co-cultivation, tomato segments were stained with the GUS substrate, and the stained area was analyzed with ImageJ^24^ (https://imagej.nih.gov/ij/). The degree of staining was categorized into four classes, and the frequency of each class was calculated. In the low-GABA tomato line, the frequency of the staining class “10% or more” was increased compared with non-transgenic tomato (Fig. 1B). The same tendency was observed in three repetitions. This result showed that inhibition of GABA accumulation during co-cultivation induced by *A. tumefaciens* inoculation increased the T-DNA transfer level.

### Introduction of the *gabT* gene into *A. tumefaciens* results in GabT activity

**Figure 2:**
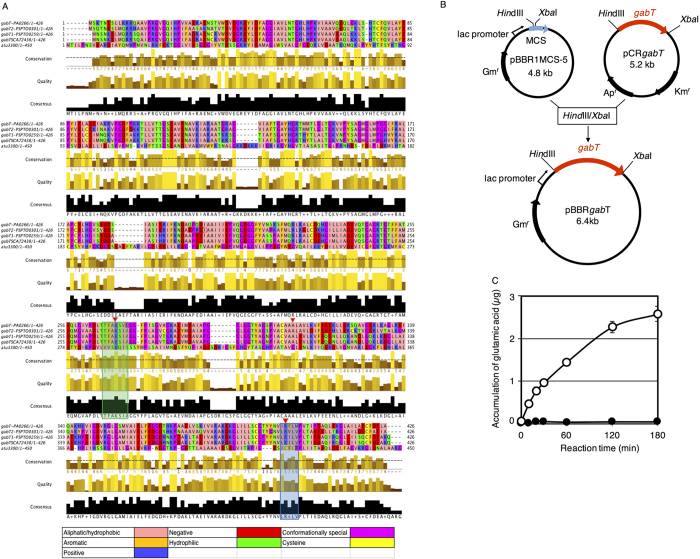
GABA transaminase activity was introduced into *A. tumefaciens*. (**A**) Amino acid sequence comparison of GABA transaminase from *E. coli* K12 (gabTSCA772438), *P. syringae* pv. tomato DC3000 (gabT1: gabT1-PSPTO0259, gabT2: gabT2-PSPTO0301), *P. aeruginosa* PAO1 (gabT-PA0266). “ClustalW2” (http://www.ebi.ac.uk/Tools/msa/clustalw2/) and “Jalview” (http://www.jalview.org) were used for calculation of amino acid multiple sequence alignment and display the alignment result, respectively. The residues were coloured according to their physicochemical properties as follows; Aliphatic/Hydrophobic, Aromatic, Positive, Negative, Hydrophilic, Conformationally Special and Cystein. Arrowheads indicate 3 of the 12 invariant amino acid residues among 16 aminotransferases with highly homologous peptides. The red box indicates a conserved motif (Ser [or Thr]-X-X-Lys) in the pyridoxalphosphate-binding peptide of aspartate aminotransferase (AAT) and histidinol-phosphate transaminases. The blue box indicates near identity between the homologous peptides of the histidinol-phosphate transaminases from *E. coli* K12 and *Saccharomyces cerevisiae*. (**B**) Construction of a plasmid for the expression of GABA transaminase (*gabT*) in *A. tumefaciens. Hin*dIII and *Xba*I fragments (ca. 1.6 kb) containing the GABA transaminase gene from *E. coli* K12 were ligated into the *Hin*dIII and *Xba*I sites of the broad-host-range plasmid pBBR1MCS-5, resulting in pBBR*gabT*. The expression of the GABA transaminase gene *gabT* was under the control of the lac promoter. MCS: multiple cloning site. (**C**) Detection of GABA activity in *A. tumefaciens*. Glutamic acid accumulation in the reaction buffer was measured according to the method of Akihiro *et al*.^48^. The open and closed circles indicate *A. tumefaciens* GV2260 (pBBR*gabT*, pIG121-Hm) and *A. tumefaciens* GV2260 (pBBR1MCS-5, pIG121-Hm), respectively. Bars represent the standard deviation (n = 3).

In this study, we used *A. tumefaciens* strain GV2260, which has the same chromosome as strain C58 33. GV2260 has the *atu3300* gene in its linear chromosome^30^. Atu3300 is predicted by Pfam (http://pfam.sanger.ac.uk) to include an Aminotrans_3 (aminotransferase class-III) domain, which is characteristic of aminotransferases^34^. The amino acid sequence of Atu3300 was compared with GabT from *E. coli* K12, *P. syringae* pv. *tomato* DC3000 and *P. aeruginosa* PAO (Fig. 2A); these GabTs display activity and function in cell growth and plant-microbial interactions^32^,^35^,^36^. Four of the GabTs (with the exception of Atu3300) were well conserved and exhibited two conserved motifs (Thr-Phe-Ala-Lys-Ser-Ile-Ala) and (Leu-Arg-Ile-Leu-Val) that correlated well with the consensus sequence of aminotransferases from *Salmonella typhimurium*^37^, *E. coli* K12, and *Saccharomyces cerevisiae*^32^. In contrast, Atu3300 did not contain these conserved motifs. Therefore, we assumed that Atu3300 shows very low or no GABA transaminase activity. The GABA accumulation induced by *A. tumefaciens* inoculation during co-cultivation inhibited T-DNA transfer (Fig. 1), which suggests that *A. tumefaciens* exhibits GabT activity and that degradation of GABA would be effective for increasing the T-DNA transfer frequency. To introduce GABA transaminase activity to *A. tumefaciens*, the GABA transaminase gene (*gabT*) was cloned from *E. coli* K12 and into the broad-host-range plasmid pBBR1MCS-5^38^, and the resulting plasmid was designated pBBR*gabT*. The *gabT* gene was expressed under the control of the *lac* promoter^21^ (Fig. 2B). Then, GabT activity was measured by monitoring glutamic acid accumulation in the reaction buffer. Only the reaction buffers containing the *A. tumefaciens* GV2260 lysate (pIG121-Hm, pBBR*gabT*) linearly increased the glutamic acid content (Fig. 2C). This result indicated that *A. tumefaciens* GV2260 exhibited no GABA transaminase activity, and we succeeded in conferring GabT activity on *A. tumefaciens* by introducing the *gabT* gene from *E. coli* K12.

### GabT activity enhances the T-DNA transfer ability of *A. tumefaciens*

**Figure 3:**
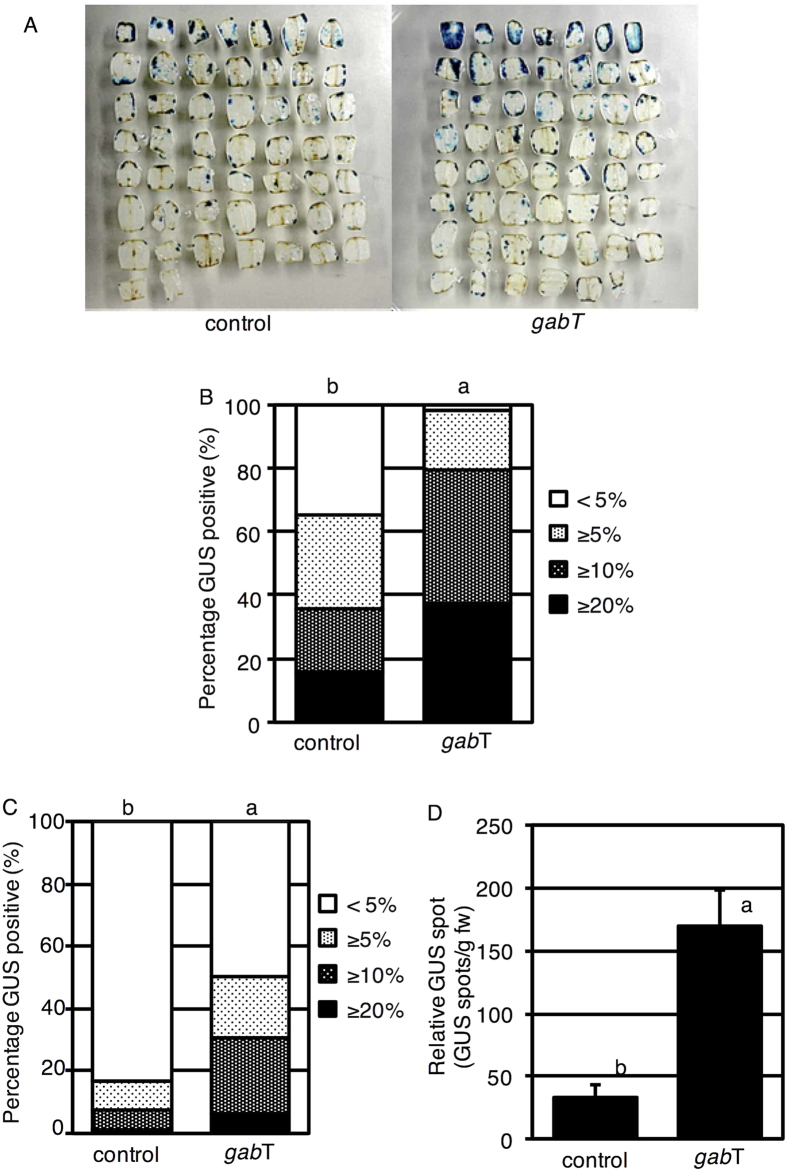
Evaluation of the transformation ability of *A. tumefaciens* with GabT. (**A**) GUS-stained explants of tomato (Micro-Tom). Explants were prepared from 7-day-old seedlings. After 3 days of co-cultivation, the explants were stained. (**B**) Estimation of T-DNA transfer for *A. tumefaciens* with GabT in Micro-Tom (tomato). GUS-stained tomato cotyledons were categorized based on the stained area, as follows: 20% or more of the area, 10% or more, 5% or more, or less than 5%. Different letters indicate significant differences according to Student’s *t*-test or the Kruskal-Wallis test; *P* < 0.01 (n = 80). (**C**) Assessment of T-DNA transfer for *A. tumefaciens* with GabT in ‘Moneymaker’. GUS-stained cotyledons were categorized as follows: 20% or more of the area, 10% or more, 5% or more, or less than 5%. Different letters indicate significant differences according to Student’s *t*-test and the Kruskal-Wallis test; *P* < 0.01 (n = 80). (**D**) Occurrence of T-DNA transformation in *E. arundinaceus*. The number of GUS-stained spots per 1 g of *E. arundinaceus* calli was counted for each treatment. The bars indicate the standard deviation (n = 3). Different letters indicate values that were significantly different according to Student’s *t*-test (*P* < 0.05). Control: *A. tumefaciens* GV2260 (pBBRMCS1–5, pIG121-Hm); *gabT: A. tumefaciens* GV2260 (pBBR*gabT*, pIG121-Hm).

To evaluate the effect of GabT on *A. tumefaciens*, two tomato cultivars (‘Micro-Tom’ and ‘Moneymaker’) and the grass *E. arundinaceus* were used. The *uidA* gene was employed as an indicator of T-DNA transfer (Fig. 3A). Compared with *A. tumefaciens* without GabT, the transformation frequency associated with a high degree of staining (10% or more) was increased approximately 2.1 or 4.0 times by the inoculation of *A. tumefaciens* carrying GabT into ‘Micro-Tom’ and ‘Moneymaker’, respectively (Fig. 3A–C). In *E. arundinaceus*, to estimate the T-DNA transfer frequency, we measured the number of blue spots on the surfaces of calli per 1 g of calli. The number of GUS spots per 1 g of calli increased four-fold when *A. tumefaciens* GV2260 (pIG121-Hm, pBBR*gabT*) was used (Fig. 3D). These results clearly showed that *A. tumefaciens* with GabT activity increased the frequency of T-DNA transfer. Therefore, degradation of GABA during co-cultivation is an effective method for increasing T-DNA transfer.

### GabT activity in *A. tumefaciens* enhances stable transformation

**Figure 4:**
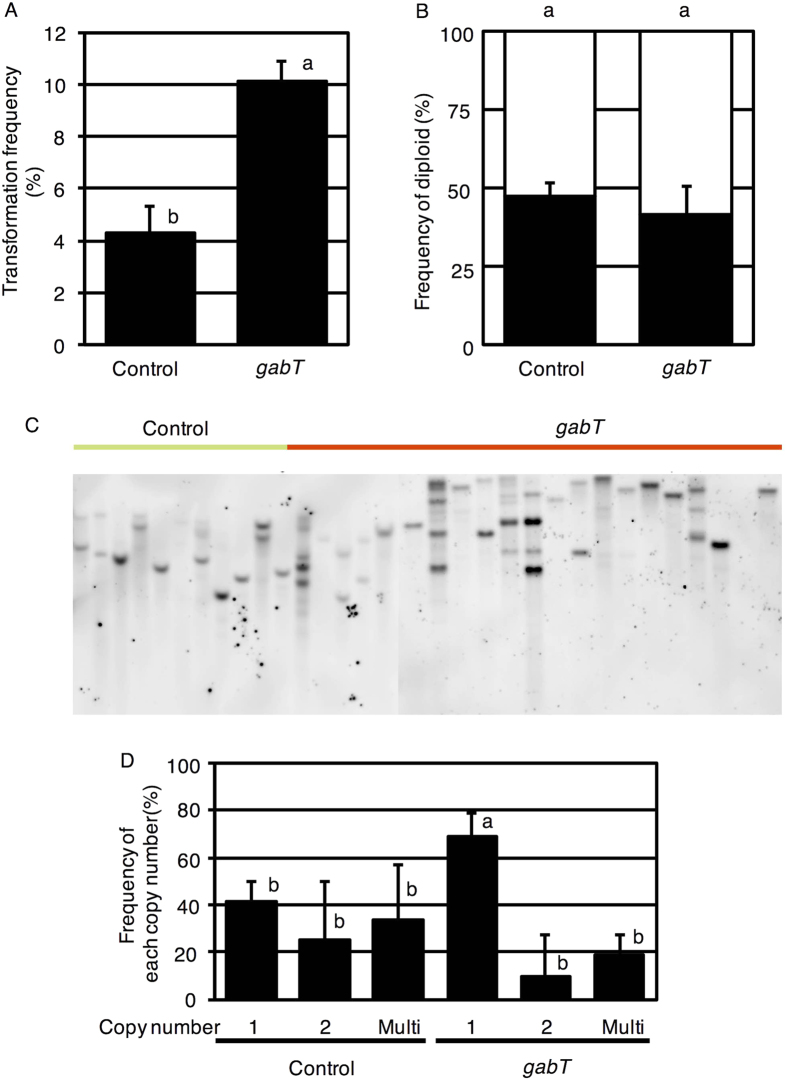
Effect of *A. tumefaciens* with GabT on stable transformation. (**A**) Effect of GabT activity in *A. tumefaciens* on stable transformation. Error bar shows the standard deviation (SD) (n = 3). Different characters indicate significant differences by Student’s *t*-test (*P* < 0.01). (**B**) Frequency of diploid appearance. Rooting shoots were checked for polyploidy. Closed and open boxes represent diploid and polyploid, respectively. Bars indicate the standard deviation (n = 3). (**C**) Southern blot analysis. (**D**) The frequency of each copy number in diploid rooting shoots. The T-DNA copy number inserted into the genome was detected through Southern blotting analysis. Bacterial strains are indicated as follows: Control: *A. tumefaciens* GV2260 (pBBR1MCS-5, pIG121-Hm); gabT: *A. tumefaciens* GV2260 (pBBR*gabT*, pIG121-Hm). Different letters indicate values that were significantly different by Tukey’s test (*P* < 0.05).

Because *A. tumefaciens* with GabT activity exhibited an increased T-DNA transfer frequency, we assumed that inoculation of the strain with GabT would also increase the frequency of stable transformation. To examine this hypothesis, almost 100 explants were inoculated with *A. tumefaciens* GV2260 (pIG121-Hm, pBBR*gabT*) or *A. tumefaciens* GV2260 (pIG121-Hm, pBBR1MCS-5). Transformation techniques were used to characterize or add gene functions to the plants. On the other hand, transformation procedure causes chromosome doubling and multi-copy insertion, which would change the phenotype, such as size, growth speed, color, and so on. Therefore, chromosome doubling and multi-copy insertion must be excluded when the phenotype or trait of transgenic plant are evaluated. The stable transformation frequency was calculated with diploid and single-copy-number plants. The stable transformation frequencies were 10.1 ± 0.7% and 4.3 ± 1.0% (mean ± SD of three repetitions using 100 explants in each experiment) (Table 1) in *A. tumefaciens* GV2260 (pIG121-Hm, pBBR*gabT*) and *A. tumefaciens* GV2260 (pIG121-Hm, pBBR1MCS-5), respectively (Fig. 4A). *A. tumefaciens* with GabT exhibited approximately 2.5 times the stable transformation frequency of *A. tumefaciens* without GabT. No significant differences in the appearance of diploidy were noted in the two types of *A. tumefaciens* (Fig. 4B), and all of the lines we obtained were independent and did not contain a cloned plant (Fig. 4C). The appearance of double and multiple copy lines was the same between the two *A. tumefaciens* strains (Fig. 4D). These results showed that *A. tumefaciens* with GabT activity exhibited an increase in stable transformation, without effects on ploidy and copy number.

## Discussion

Our results showed that *A. tumefaciens* infection increased the GABA contents of tomato cotyledon segments during co-cultivation. Based on this result, we assumed that the GABA accumulated during co-cultivation affected T-DNA transfer. To test this hypothesis, we used the GAD RNAi tomato, which showed a low GABA level during co-cultivation, and *A. tumefaciens* with the ability to degrade GABA. Compared with the control, the T-DNA transfer frequency was increased in the GAD RNAi tomato line (Fig. 1B). *A. tumefaciens* with GabT activity (Fig. 2B) also increased the T-DNA transfer in ‘Micro-Tom’, ‘Moneymaker’, and *E. arundinaceus* (Fig. 3). The frequency of stable transformation was also increased in ‘Micro-Tom’ (Table 1). These results clearly show that the GABA accumulated during co-cultivation inhibited both T-DNA transfer and stable transformation.

The present study clearly showed that GABA that accumulates during co-cultivation, induced by inoculation with *A. tumefaciens*, inhibits T-DNA transfer (Figs 1 and 3). These results suggest that GABA affects an early step of the *Agrobacterium*-plant interaction. On the other hands, previous studies conclude that GABA plays a role in tumors at a later stage of the *Agrobacterium*-plant interaction, and not in earlier stages^26^. These inconsistent results might be attributed to differences in the applied evaluation of T-DNA transfer methods, as in the previous study, T-DNA transfer was evaluated based on the proportion of stained leaf discs per infection^26^, whereas we evaluated the frequency of T-DNA transfer in each tomato segment or 1 g of *E. arundinaceus* calli. These methods allowed us to evaluate the T-DNA transfer in detail. Based on these results, we concluded that the high accumulation of GABA induced by *A. tumefaciens* infection inhibits T-DNA transfer and that GABA is involved in both the early and later stages of the *Agrobacterium*-plant interaction.

The genome of *A. tumefaciens* strain C58 encodes *atu3300* in its linear chromosome^30^. This gene has 68.1% similarity and 23.7% identity with GABA transaminase from *E. coli* (GabT). Atu3300 included an aminotransferase class-III and was predicted to have GabT activity. However, although Atu3300 has an aminotransferase class-III domain, it did not show GabT activity (Fig. 2C). In comparisons of GabT with several aspartate, tyrosine, and histidinol-phosphate transaminases, previous studies predicted that two motifs, containing Lys268 and Arg398, are involved in active-site formation in the GabT protein^32^,^39^. The GabT proteins from *E. coli* K12, *P. syringae* pv. tomato DC3000 and *P. aeruginosa* PAO have these motifs. However, Atu3300 does not contain these motifs (Fig. 2A). According to the present study and previous work, the lack of GabT activity in *A. tumefaciens* appears to result from the lack of these motifs in Atu3300 (Fig. 2A), indicating that the two motifs in GabT containing Lys268 and Arg398 are essential for this activity.

Removal of GABA is more effective than *vir* gene stimulation. Our results indicated that the reduction of GABA during co-cultivation increased T-DNA transformation under high *vir* gene expression induced by 200 μM acetosyringone (Figs 1B and 3B,C,D), which is sufficient to fully induce the *vir* gene^20^. Although the possibility that GABA is involved in *vir* gene expression cannot be discarded because of two contradictory results^26^,^28^, our results show that GABA has a stronger effect on T-DNA transfer than stimulation of *vir* gene expression. As the QS signal accumulates in tumor, which is a late stage of the *Agrobacterium*-plant interaction^40^, GABA should inhibit T-DNA transfer independently of a QS signal in an early stage. This conclusion suggests the existence of an “unknown pathway” independent of the QS signal. The unknown pathway involved in GABA function might have a stronger influence on T-DNA transformation.

Depending on the species or cultivar, the effect of inhibiting negative factors seems to differ. In ‘Moneymaker’ and *E. arundeinaceus*, the new *A. tumefaciens* strain with GabT activity is more effective than the previously used strain that inhibits ethylene during co-cultivation^24^. In addition to GABA^25^,^26^ and ethylene^17^,^18^,^19^,^20^, salicylic acid, cytokinin, auxin and abscisic acid are also known negative factors in the *Agrobacterium*-plant interaction^41^,^42^,^43^,^44^. Selecting negative factors based on the species involved might be important for increasing transformation and broadening host ranges. Moreover, removing multiple such negative factors would have an effect on the transformation frequency and adaptation to a wide range of host plants.

We succeeded in producing an *A. tumefaciens* strain with improved potential for transformation by imbuing it with the ability to remove GABA, which is a negative factor in the *Agrobacterium*-plant interaction. Removal of GABA increased the transformation frequency approximately 2.5 times. Therefore, this newly bred bacterium enables us to decrease the number of cotyledons used for transformation by 60%. Thus, this strain allows us to reduce the time and the labor required for transformation. Based on this result, we conclude that this new strain might be a useful tool for plant genetic engineering.

For plant molecular breeding, the genetic modification technique is very important, and *Agrobacterium*-mediated transformation is the most frequently used method. Therefore, substantial effort has been expended to adapt *Agrobacterium*-mediated transformation to a wide variety of plants. However, species and genotypes recalcitrant to genetic transformation still exist, and the improvement has been required. Transformation process include the three steps: first step is T-DNA transfer, second step is selection of transgenic cells, and the third step is regeneration from the transgenic cells. Previous study indicated japonica rice showed higher transformation frequency than indica rice. Comparing with japonica rice, T-DNA transfer frequency was very low in indica rice^45^. This result suggested that improvement of this process was effective. Indeed, improvement of T-DNA transfer increased stable transformation^46^,^47^. Inevitable, we focused on improvement of T-DNA transfer through new bred *A. tumefaciens* strain. Our new bred *A. tumefaciens* strain with GABA degradation ability improved T-DNA transfer in two genotypes of tomato and *E. arundinaceus*, and increased stable transformation in tomato ‘Micro-Tom’. We succeeded in producing an *A. tumefaciens* strain with improved potential for transformation by imbuing it with the ability to degrade GABA. The strain created in this study might represent a new system for improving the transformation frequency in recalcitrant species and genotypes.

## Materials and Methods

### Bacterial strains and culture conditions

*E. coli* K12 and DH5α were grown at 37 °C in Luria Broth (LB) medium (1% bacto-tryptone, 0.5% yeast extract, and 0.5% NaCl). *A. tumefaciens* strain GV2260, a derivative of the C58 strain, was grown at 28 °C in LB medium. Antibiotics were added at the following final concentrations: ampicillin at 100 μg ml^−1^ for *E. coli* K12 and *A. tumefaciens* and gentamicin at 50 μg ml^−1^, kanamycin at 50 μg ml^−1^, and spectinomycin at 50 μg ml^−1^ for *A. tumefaciens*.

### *A. tumefaciens* growth conditions

*A. tumefaciens* GV2260 was cultured on solid LB medium at 28 °C for 2 days. A single colony was then picked and cultured in 2 ml of LB medium at 28 °C at 200 rpm for 2 days until the pre-culture reached the stationary phase. After pre-culture, 15 μl of culture medium was added to 15 ml of LB medium containing antibiotics, and culturing was continued for 20–22 hours at 28 °C, with shaking at 200 rpm. After the culture reached an OD_600_ of 0.8–1.0, to collect bacterial cells, the culture was centrifuged at 3,000 × *g* for 10 min.

### Construction of the *gabT* expression plasmid

The *gabT* gene was cloned from the genome of *E. coli* K12 via polymerase chain reaction (PCR) using the primers gabTF (5′-aagcttaatgaacagcaataaagagtt-3′) and gabTR (5′-tctagactactgcttcgcctcatcaaaac-3′). The 1294-bp amplified fragment was inserted into the pCRTOPO vector (Invitrogen, Carlsbad, CA, USA) to generate pCR*gabT*. We checked the sequence using an ABI Sequence Analyzer (Applied Biosystems, MA, USA), and the sequence was found to be identical to accession number 6061113 in the National Center for Biotechnology Information (NCBI, http://www.ncbi.nlm.nih.gov) database. The *gabT* fragment was subcloned into the multiple cloning site of the broad-host-range plasmid pBBR1MCS-5^38^ using *Hin*dIII and *Xba*I (New England Biolabs, Hirchin, UK) to generate a *lacZ::gabT* translational fusion (pBBR*gabT*) (Fig. 2B).

### GabT activity in *A. tumefaciens*

A pellet of *A. tumefaciens* cells was re-suspended in 100 μl of BugBuster Master mix (Novagen, MA, USA) for lysate preparation. The protein concentration of the lysate was measured by a BCA Protein Assay Kit (Novagen, MA, USA). The protein content was adjusted to 100 μg per reaction mixture. The reaction mixture contained 0.1 M bicine-NaOH, 0.1 M pyridoxal phosphate, 10 mM 2-ketoglutarate, 10 mM GABA, and a protease inhibitor cocktail. The reaction mixture was incubated at 37 °C for 0, 10, 20, 30, 60, 120 or 180 min. GabT metabolizes GABA to glutamate; therefore, to estimate GabT activity, we detected the glutamate concentration in the reaction mixture using a Yamaki glutamate assay kit (Yamaki, Tokyo, Japan)^48^.

### Plant material

Non-transgenic tomato seeds (*Solanum lycopersicum* ‘Micro-Tom’ or ‘Moneymaker’) and a *GAD*-suppressed ‘Micro-Tom’ transgenic line (*RNAi-SlGADall*) that we used in a previous study^29^ were employed in this study. ‘Moneymaker’ exhibits medium-sized fruits and is a commercialized cultivar. The seeds were washed with 70% ethanol for 10 seconds, sterilized with 5% hypochlorous acid containing 10% Triton X-100 for 45 min, and washed three times with sterilized water. After the third wash, the seeds were kept in water for 2 days. The sterilized tomato seeds were sown on Murashige and Skoog (MS) medium^49^ containing 15 g l^−1^ sucrose (Wako, Tokyo, Japan) and 0.3% Gelrite (Wako, Tokyo, Japan) and then grown for 7 days.

Calli of *E. arundinaceus*, known as a high biomass producer, were kindly provided by Prof. Masahiro Mii of Chiba University, Japan. The calli induced from the seeds on MS medium containing 1 g l^−1^ casamino acids, 2 mg l^−1^ 2,4-dichlorophenoxyacetic acid (2, 4-D), 0.2 mg l^−1^ 6-benzylaminopurine (BAP), 30 g l^−1^ 4-O-α-D-glycopyranosyl-D-glycopyranose (maltose H) (Wako, Tokyo, Japan) and 0.3% Gelrite were subcultured for 2 weeks before *A. tumefaciens* inoculation.

### Measurement of GABA content

To measure the GABA content, tomato seedlings and cotyledon segments were prepared. Approximately 50 mg of powdered sample was added to 500 μl of 8% (w/v) trichloroacetic acid and mixed via vortexing for 30 sec. The mixture was subsequently centrifuged at 10,000 × *g* for 20 min at 4 °C, and 300 μl of the supernatant was transferred to a new tube and mixed vigorously with 400 μl of pure diethyl ether for 10 min. The solution was then centrifuged at 10,000× *g* for 10 min at 4 °C, after which the upper phase of the diethyl ether was removed and the previous step was repeated. After centrifugation again at 10,000× *g* for 10 min at 4 °C, the upper phase was removed. To completely remove the remaining diethyl ether, the samples were incubated under a draft of air for 30 min. A 30 μl aliquot of the lower phase was transferred to a new 1.5 ml tube and dried using an evaporator (CVE3100, TOKYO RIKAKIKAI, Tokyo, Japan). The dried samples were washed with 150 μl of sterile distilled water. After washing, the samples were dissolved in 0.1 N HCl for amino acid analysis (JLC-500/V2, Japan Electron Optics Laboratory, Tokyo, Japan).

### *Agrobacterium*-mediated T-DNA transfer

Bacterial cells were re-suspended in liquid MS medium containing 30 g l^−1^ glucose and 200 μM acetosyringone (Wako, Tokyo, Japan) at pH 5.2, and the OD_600_ was adjusted to 0.4–0.5. Cotyledons of 7-day-old tomato seedlings were cut into four pieces and subjected to inoculation with *A. tumefaciens*. Eighty explants were subjected to each treatment. The inoculated explants were cultured on co-cultivation medium (pH 5.2) containing MS salts, 30 g l^−1^ glucose, 200 μM acetosyringone and 0.3% Gelrite (Wako, Tokyo, Japan) at 25 °C for 3 days in the dark. After 3 days of co-cultivation, the tomato explants were assayed histochemically for β-glucuronidase (GUS) activity using GUS staining solution containing 100 mM NaPO_4_, 10 mM EDTA, 2.5 mM potassium ferrocyanide, 2.5 mM potassium ferrocyanide, 0.1% Triton X-100, and 0.5 mg ml^−1^ X-Gluc.

Calli of *E. arundinaceus* that had been subcultured for 2 weeks were also inoculated with *A. tumefaciens.* After co-cultivation, the GUS activity of *E. arundinaceus* calli was histochemically assayed with GUS staining solution, as described above.

### Stable tomato transformation

After 3 days of co-cultivation, ‘Micro-Tom’ cotyledon segments were placed on callus-induction medium (MS medium containing 0.3% Gelrite, 1.5 mg l^−1^ zeatin, 100 mg l^−1^ kanamycin, and 375 mg l^−1^ augmentin [GlaxoSmithKline, MDX, UK]) for 4 weeks. Calli that formed from the segments were cultured on shoot-induction medium (MS medium containing 0.3% Gelrite, 1.0 mg l^−1^ zeatin, 100 mg l^−1^ kanamycin, and 375 mg l^−1^ augmentin) for 4 weeks. The shoots were then placed on rooting medium, which consisted of half-strength MS medium, 0.3% Gelrite (Wako, Tokyo, Japan), 100 mg l^−1^ kanamycin, and 375 mg l^−1^ augmentin, for 2 weeks. Tissues were subcultured every 10–14 days. The ploidy of the rooting shoots was checked via flow cytometry.

### Ploidy analysis

One square centimeter of leaf was cut from the rooting shoots and chopped in 250 μl of nucleus-extraction solution (CyStain UV Precise P, Sysmex, Hyogo, Japan). To purify the nucleus extraction solution, 1 mm^2^ mesh was used. After purification, 1 ml of staining solution (CyStain UV Precise P, Sysmex, Hyogo, Japan) was added, followed by incubation for 1 min. This solution was applied to an Attune focusing analyzer (ABI, MA, USA), and diploid plants were selected. The diploid plants were planted on solid medium and acclimatized.

### Southern blot analysis

Genomic DNA was extracted from young tomato leaves using the Maxwell 16 System DNA Purification kit (Promega, WI, USA). The purified DNA was digested with *Hin*dIII, then electrophoretically separated in a 0.8% agarose gel and transferred to Gene Screen Plus nylon membranes (Roche Diagnostics, Basel, Switzerland) with 20 × saline-sodium citrate buffer. After ultraviolet cross-linking, the membranes were hybridized in a solution containing 7% sodium dodecyl sulfate, 50% deionized formamide, 50 mM sodium phosphate (pH 7.0), 2% blocking solution, 0.1% N-lauroylsarcosine, 0.75 M NaCl, and 75 mM sodium citrate at 42 °C overnight. For hybridization, a digoxigenin (DIG)-labeled DNA probe specific for *npt*II (0.8 Kb) was used. A DIG-labeled probe was generated using DIG-High Prime, and the DIG signal was detected according to the manufacturer’s protocol (Roche Diagnostics, Basel, Switzerland).

## Additional Information

**How to cite this article**: Nonaka, S. *et al*. An *Agrobacterium tumefaciens* Strain with Gamma-Aminobutyric Acid Transaminase Activity Shows an Enhanced Genetic Transformation Ability in Plants. *Sci. Rep.* **7**, 42649; doi: 10.1038/srep42649 (2017).

**Publisher's note:** Springer Nature remains neutral with regard to jurisdictional claims in published maps and institutional affiliations.

## Figures and Tables

**Table 1:** Transformation frequency in Tomato ‘Micro-Tom’.

	Treatment	Number				Ratio (%)	
		Segments	Rooting	Diployd Shoot	Singl Copy Shoot	Diployd Shoot/Rooting	Single Copy Shoot/Segments
First Experiment	Control	140	12	6	6	50.0	4.3
	*gabT*	156	29	15	15	51.7	9.6
Second Experiment	Control	120	8	4	4	50.0	3.3
	*gabT*	102	42	15	10	35.7	9.8
Third Experiment	Control	93	14	6	5	42.9	5.4
	*gabT*	91	43	16	10	37.2	11.0

Control and *gabT* mean inoculation with *A. tumefaciens* GV2260 (pIG121-Hm, pBBR1MCS-5) and *A. tumefaciens* GV2260 (pIG121-Hm, pBBR*gabT*), respectively.
